# Serum Tumor Necrosis Factor-*α* and Interferon-*γ* Levels in Pediatric *Mycoplasma pneumoniae* Pneumonia: A Systematic Review and Meta-Analysis

**DOI:** 10.1155/2018/8354892

**Published:** 2018-09-10

**Authors:** Ying Wang, Yongsheng Zhang, Wenting Lu, Liying Wang

**Affiliations:** ^1^Institute of Pediatrics, The First Hospital of Jilin University, Changchun, Jilin 130021, China; ^2^Centre for Reproductive Medicine and Center for Prenatal Diagnosis, The First Hospital of Jilin University, Changchun, Jilin 130021, China; ^3^Department of Molecular Biology, Norman Bethune College of Medicine, Jilin University, Changchun, Jilin 130021, China

## Abstract

**Background:**

*Mycoplasma pneumoniae* pneumonia (MPP) is one of the most common forms of community-acquired pneumonia in children. The objective of this study was to explore potential changes in levels of serum tumor necrosis factor-*α* (TNF-*α*) and interferon-*γ* (IFN-*γ*) associated with pediatric MPP.

**Methods:**

This protocol has been registered (PROSPERO 2017: CRD42017077979). A literature search was performed in October 2017 using PubMed, Embase, the Cochrane Library, and other Chinese medical databases to identify studies. The meta-analysis was performed using Review Manager 5.3 software. Random-effect models were used to estimate mean differences (MDs) and 95% confidence intervals (CIs) of cytokine levels.

**Results:**

Twelve studies were included in the meta-analysis, encompassing 2,422 children with MPP and 454 healthy control children. Serum TNF-*α* levels were significantly higher in children with MPP compared with healthy children (MD = 22.5, 95% CI = 13.78–31.22, *P* < 0.00001), and there was significant heterogeneity across studies (*I*^2^ = 100%, *P* < 0.00001). Subgroup analyses showed no evidence for a difference in serum TNF-*α* levels between children with refractory and nonrefractory MPP. Serum IFN-*γ* levels did not significantly differ in children with MPP compared with healthy children (MD = 4.83, 95% CI = −3.27–12.93, *P*=0.24).

**Conclusions:**

Our meta-analysis showed that serum TNF-*α* and IFN-*γ* levels were significantly elevated and unchanged, respectively, in pediatric MPP. Because infection by different pathogens has variable effects on serum TNF-*α* and IFN-*γ* levels, the finding could be helpful in developing novel diagnostic methods.

## 1. Introduction

*Mycoplasma pneumoniae* (MP) is one of the most prevalent etiological agents of community-acquired pneumonia in children [1, 2]. Infection by MP is traditionally thought to be self-limiting [3], but under some circumstances, it can result in severe life-threatening diseases such as acute respiratory distress syndrome, necrotizing pneumonitis, and fulminant pneumonia. The incidence of MP pneumonia (MPP) has been increasing in recent years [2]. However, symptoms and radiographic findings in children with MPP are often similar to those associated with other respiratory infections [4]. Thus, early diagnosis of MPP is crucial for initiating appropriate antibiotic therapy. Proinflammatory cytokines play an important role in the mechanism of MP infection [5–7].

The proinflammatory cytokine tumor necrosis factor-alpha (TNF-*α*) is an important initiator of inflammatory and bactericidal processes [8, 9]. In bacterial pneumonia, macrophage-derived TNF-*α* is elevated, resulting in recruitment of inflammatory cells to sites of infection [8, 10, 11]. However, TNF-*α* may not play a significant antiviral role, and its levels in serum do not change significantly during viral pneumonia [8, 12, 13]. Serum levels of TNF-*α* in patients with MPP are less well defined, with some studies showing elevated levels and others no difference [14, 15]. Another proinflammatory cytokine, interferon-gamma (IFN-*γ*), is a critical mediator of antiviral immunity and is often associated with chronic inflammation [16, 17]. Serum levels of IFN-*γ* are typically elevated during both viral and bacterial infections [18, 19], but its levels in pediatric MPP patients have been inconsistent across studies [20, 21].

Thus, the purpose of this meta-analysis was to analyze levels of serum TNF-*α* and IFN-*γ* in pediatric MPP and compare these with levels in healthy children.

## 2. Methods

### 2.1. Literature Search and Study Selection

A literature search was performed in October 2017 using PubMed, Embase, the Cochrane Library, and other Chinese medical databases to identify studies. The following search terms were used: (“cytokine”) or (“tumor necrosis factor-*α*” or “TNF-*α*”) or (“interferon-*γ*” or “IFN-*γ*”) and (“*Mycoplasma pneumoniae* pneumonia” or “MPP”). The searches were restricted to studies whose subjects were children; no language restrictions were applied. The reference lists and supplemental materials associated with the search results were manually inspected to identify additional relevant publications. A meta-analysis of the mean differences (MDs) of serum TNF-*α* and IFN-*γ* levels in pediatric MPP was undertaken. The Prospero registration number is CRD42017077979.

**Figure 1:**
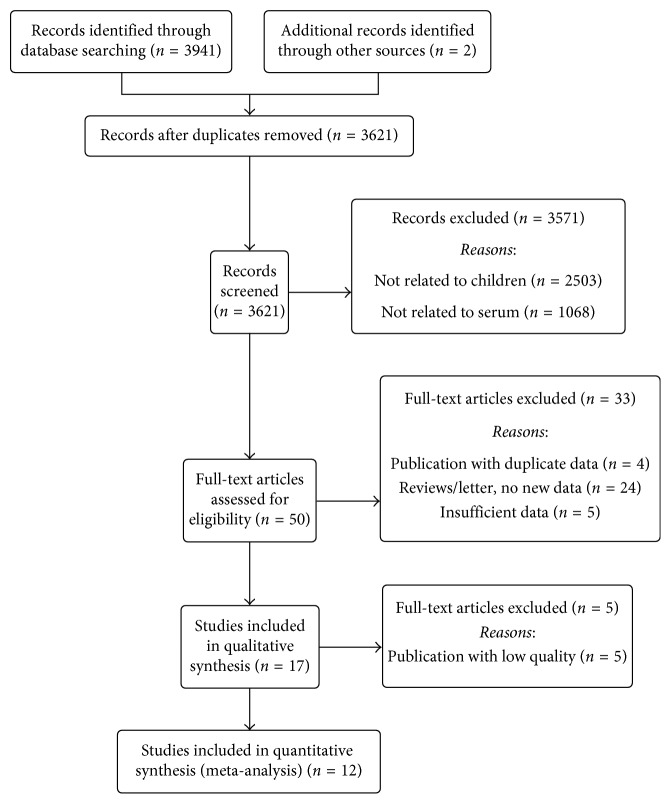
Flow diagram of included studies for this meta-analysis.

All studies aiming to explore the association between serum TNF-*α* and IFN-*γ* levels and pediatric MPP were included. The study inclusion criteria were as follows: (i) study subjects included children with MPP; (ii) study subjects included a group of healthy control children; (iii) serum TNF-*α* and IFN-*γ* levels were measured; and (iv) full text, original research articles could be obtained. The study exclusion criteria were as follows: (i) lack of data on serum TNF-*α* and IFN-*γ* levels in pediatric MPP; (ii) not a primary study or not a case-control design; (iii) insufficient data extracted from the articles or the full text could not be obtained; and (iv) duplicate studies. The study inclusion and exclusion procedures are summarized in Figure 1.

### 2.2. Data Extraction

Two investigators (Y Wang and YS Zhang) independently performed the data extraction. The general characteristics of the study were extracted using a standardized data extraction form on which publication information (first author's name, publication year, and country) and subject characteristics (serum cytokine measurement method, MPP type, control type, and sample size) were noted. If no standard deviations for serum TNF-*α* and IFN-*γ* concentrations were available, these values were calculated using confidence intervals and medians [22]. If multiple published reports of the same study population were available, we included only the report with the largest sample size and the most complete data. Discrepancies were resolved by discussion with other investigators (WT Lu and L Wang).

### 2.3. Statistical Analysis

The meta-analysis was performed using Review Manager 5.3 software. Serum TNF-*α* and IFN-*γ* levels were extracted as the means ± standardized deviations (SDs) of each study. MDs with 95% CIs were used to determine the strength and directionality of the association between serum TNF-*α* and IFN-*γ* levels and pediatric MPP. The pooled MDs for TNF-*α* and IFN-*γ* concentrations associated with pediatric MPP were calculated. Subgroup analyses were performed to compare levels in children with refractory and nonrefractory MPP. Heterogeneity was assessed using Cochran's *Q* test and the I-squared statistic. If *P* < 0.1 or *I*^2^ > 50%, heterogeneity was considered significant, and a random-effect model was used.

## 3. Results

### 3.1. Search Results

The steps for screening and the study selection procedure are presented in Figure 1. A total of 3,621 relevant articles were initially identified from PubMed, Embase, the Cochrane Library, and other Chinese medical databases. Through screening of titles and abstracts, 50 publications met the study inclusion criteria. After reading the full text, 12 studies were included in the meta-analysis [14, 15, 20, 21, 23–30].

### 3.2. Levels of Serum TNF-*α* and IFN-*γ* in Pediatric MPP

**Figure 2:**
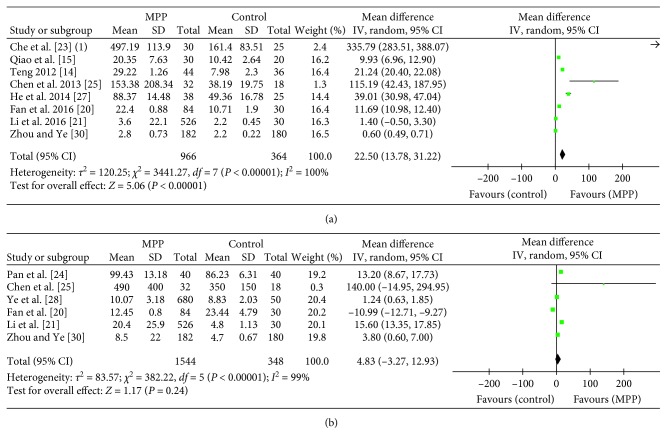
Forest plots showing mean difference (MD) and confidence intervals (CI) of the serum TNF-*α* and IFN-*γ* levels to diagnose pediatric MPP. (a) TNF-*α* and (b) IFN-*γ* related results have been shown.

The results of 12 studies of serum TNF-*α* and IFN-*γ* levels in children with MPP are summarized in Table 1. The pooled MDs revealed that serum TNF-*α* levels were higher in the pediatric MPP group as compared with age-matched healthy controls (MD = 22.5, 95% CI = 13.78–31.22, *P* < 0.00001) (Figure 2(a)). Substantial heterogeneity was observed among studies (*I*^2^ = 100%; *P* < 0.00001). As shown in Figure 2(b), serum IFN-*γ* levels did not significantly differ between children with MPP and healthy control children (MD = 4.83, 95% CI = −3.27–12.93; *P*=0.24), with significant heterogeneity across studies (*I*^2^ = 99%; *P* < 0.00001).

### 3.3. Subgroup Analysis

**Figure 3:**
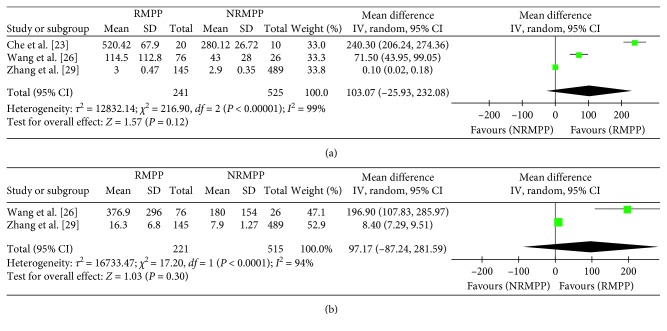
Forest plots analysis change rule in the serum TNF-*α* and IFN-*γ* levels between refractory and nonrefractory pediatric MPP. (a) TNF-*α* and (b) IFN-*γ* related results have been shown.

Although the result above demonstrated that serum TNF-*α* levels significantly differed between children with MPP and healthy children, levels in different pediatric MPP subgroups were still unknown. We found that serum TNF-*α* and IFN-*γ* levels were not significantly different in children with refractory and nonrefractory MPP (TNF-*α*: MD = 103.07, 95% CI = −25.93–232.08, *P*=0.12; IFN-*γ*: MD = 97.17, 95% CI = −87.24–281.59, *P*=0.30) (Figure 3). In the subgroup analysis, significant heterogeneity was observed both for TNF-*α* levels (*I*^2^ = 99%; *P* < 0.00001) and IFN-*γ* levels (*I*^2^ = 94%; *P* < 0.00001) (Figure 3).

### 3.4. Sensitivity Analysis and Reporting Bias

Sensitivity analysis was performed by excluding studies one by one. No obvious changes were found in the results, which confirmed their stability. We did not construct a funnel plot as they are known to be unreliable when constructed using fewer than 10 studies [31].

## 4. Discussion

TNF-*α* is involved in normal inflammatory reactions and immune responses and is crucial for bactericidal processes [10, 11]. Serum TNF-*α* levels are usually elevated in bacterial pneumonia and not significantly altered in viral pneumonia [8, 12, 13]. Our results revealed that serum TNF-*α* levels were higher in children with MPP compared with healthy controls. This finding suggests that a large amount of TNF-*α* is released systemically in children infected by MP to combat the pathogen via inflammatory processes. We speculate that MP and other bacteria use similar signals to promote inflammatory responses. Previously, it was reported that the actin-like protein of MP and other bacterial species might share a common evolutionary ancestor [32]. Due to the limited study of serum TNF-*α* levels in different pediatric MPP subgroups, we could not obtain sufficient data to perform a rigorous statistical analysis; this may be one explanation for our finding that serum TNF-*α* levels did not differ in refractory and nonrefractory MPP.

IFN-*γ* is a major mediator of antiviral immunity and inflammatory responses [16, 17]. Serum IFN-*γ* levels are consistently elevated in both viral and bacterial pneumonia [18, 19]. In our meta-analysis, serum IFN-*γ* levels were not significantly different in children with MPP and healthy controls. We speculate that IFN-*γ* may be produced predominantly at sites of local inflammation rather than systemically. Consistent with this hypothesis, some studies have reported elevated IFN-*γ* levels in sputum and alveolar lavage fluid of children with MPP [33, 34]. Our results showed that serum levels of TNF-*α* and IFN-*γ* during infection with MP differed from those observed during infection by other bacterial and viral pathogens causing pneumonia. Thus, we speculate that serum TNF-*α* and IFN-*γ* levels in pediatric MPP may be potential diagnostic markers of infection by this pathogen.

The main limitation of this study was the significant heterogeneity observed across included studies. This heterogeneity remained in subgroup analyses of children stratified by disease severity, which indicated that variation in test methods, measurement reagents, or MPP onset time might be potential sources. Of the 12 included studies, cytokine testing was performed using radioimmunoassay, flow cytometry, and ELISA in 1, 4, and 7 studies, respectively (Table 1). Each measurement method yielded different cutoff values, which may have contributed to greater heterogeneity. Moreover, even for the same measurement method (such as ELISA), reagents were purchased from different manufacturers in each study; the reagents used may have affected cutoff values, also contributing to heterogeneity. Early-onset or late-onset MPP can induce varying degrees of inflammatory responses, and thus variation in time of testing could also introduce heterogeneity. Finally, most of the included studies had relatively small sample sizes. Further studies with larger sample size are needed to reduce statistical heterogeneity in TNF-*α* and IFN-*γ* levels and improve their prognostic value as diagnostic biomarkers for MPP, as well as to make meta-analyses more valuable. Due to the limited number of included studies, we did not attempt to analyze covariates as possible sources of heterogeneity. Although significant heterogeneity was detected, sensitivity analysis showed that no single study influenced the pooled results.

In conclusion, although few studies have analyzed serum TNF-*α* and IFN-*γ* levels in pediatric MPP, our meta-analysis showed that serum TNF-*α* levels in children with MPP were higher than those in healthy children. However, serum IFN-*γ* levels were not different in children with MPP and healthy controls. Based on the varying trends of serum TNF-*α* and IFN-*γ* levels during infection by different pathogens, this finding may be useful for distinguishing pathogens in clinical settings.

## Figures and Tables

**Table 1:** Characteristics of studies included in the meta-analysis.

Author/year of publication	Country	Plasma cytokines assay method	*Mycoplasma pneumoniae* pneumonia group				Control group			
			Total *N*	Source of pneumonia type	TNF*α* (pg/ml)	IFN*γ* (pg/ml)	Total *N*	Source of control	TNF*α* (pg/ml)	IFN*γ* (pg/ml)
Che et al. [23] (1)	China	Radioimmunoassay	30	MPP	497.19 ± 113.90	—	25	Healthy	161.40 ± 83.51	—
Che et al. [23] (2)	China	Radioimmunoassay	20	RMPP	520.42 ± 67.90	—	10	NRMPP	280.12 ± 26.72	—
Pan et al. [24]	China	ELISA	40	MPP	—	99.43 ± 13.18	40	Healthy	—	86.23 ± 6.31
Teng [14]	China	ELISA	44	MPP	29.22 ± 1.26	—	36	Healthy	7.98 ± 2.30	—
Qiao et al. [15]	China	ELISA	30	MPP	20.35 ± 7.63	—	20	Healthy	10.42 ± 2.64	—
Chen et al. [25]	China	ELISA	32	MPP	153.38 ± 208.34	490 ± 400	18	Healthy	38.19 ± 19.75	350 ± 150
Wang et al. [26]	China	ELISA	76	RMPP	114.5 ± 112.8	376.9 ± 296	26	NRMPP	43 ± 28	180 ± 154
He et al. [27]	China	ELISA	38	MPP	88.37 ± 14.48	—	25	Healthy	49.36 ± 16.78	—
Ye et al. [28]	China	Flow cytometry	680	MPP with acute phase	—	10.07 ± 3.18	50	Healthy	—	8.83 ± 2.03
Zhang et al. [29]	China	Flow cytometry	145	RMPP	3.0 ± 0.47	16.3 ± 6.8	489	NRMPP	2.9 ± 0.35	7.9 ± 1.27
Fan et al. [20]	China	ELISA	84	MPP	22.4 ± 0.88	12.45 ± 0.8	30	Healthy	10.71 ± 1.90	23.44 ± 4.79
Li et al. [21]	China	Flow cytometry	526	MPP	3.6 ± 22.1	20.4 ± 25.9	30	Healthy	2.2 ± 0.45	4.8 ± 1.13
Zhou and Ye [30]	China	Flow cytometry	182	MPP	2.8 ± 0.73	8.5 ± 22.0	180	Healthy	2.2 ± 0.22	4.7 ± 0.67

RMPP: refractory *Mycoplasma pneumoniae* pneumonia; NRMPP: nonrefractory *Mycoplasma pneumoniae* pneumonia.
